# Detection of high-risk human papillomavirus genotypes 58 and 59 among oral squamous cell carcinoma patients

**DOI:** 10.1186/s13027-025-00687-7

**Published:** 2025-07-29

**Authors:** Snigdha Maity, Sreeraj Surendran, Prachi Malasane, Ujwal Shetty, Rithesh K. B., Priyanka Shetty, Prahlad Shetty, Monisha J. Shetty, Nikitha S., V. Vaishnavi, Chiranjay Mukhopadhyay, Vijaya Hegde, Anitha Jagadesh

**Affiliations:** 1 Manipal Institute of Virology, Manipal Academy of Higher Education, Manipal, India; 2 A. J. Research Center, A. J. Institute of Medical Sciences and Research Centre, Mangalore, Karnataka India; 3 Oral and Maxillofacial Surgery, A. J Institute of Dental Sciences, Mangalore, Karnataka India; 4 Oral Pathology, A. J Institute of Dental Sciences, Mangalore, Karnataka India; 5 Public Health Dentistry, A. J Institute of Dental Sciences, Mangalore, Karnataka India

**Keywords:** Oral squamous cell carcinoma, Human papillomavirus, Cancer, HPV 58, HPV 59

## Abstract

Oropharyngeal squamous cell carcinoma (OPSCC), a type of head and neck cancer (HNC), represents a major global health issue contributing to substantial morbidity and mortality. Human papillomavirus (HPV) is an established oncogenic virus and is among the major causes for OPSCC. Although HPV has been identified as a risk factor for oral squamous cell carcinoma (OSCC). Limited information exists on its current prevalence and associated risk factors in India.

The current research aimed to detect different high-risk HPV genotypes among OSCC and OPSCC patients attending a tertiary care hospital in Mangalore, India. After consenting to participate in the study, tumor tissue biopsies were collected from 25 oral cancer patients. Nucleic acid was extracted from samples and tested for high-risk HPV by real-time PCR and conventional multiplex PCR. Furthermore, Sanger sequencing and bioinformatic analysis were performed to identify the specific genotypes. Among the 25 biopsy samples tested, three samples (12%) were positive for high-risk HPV. The sequencing results indicated that two of the samples belonged to HR HPV type 58, and one belonged to type 59. Clinical analysis revealed a significant association between HPV-positive OSCC and high alcohol consumption and tobacco chewing.

The findings of the present study suggest that in addition to traditional risk factors such as alcohol and tobacco use, HPV may also be a risk factor for the development and progression of OSCC, although its specific etiological role remains unclear. While most Indian studies have consistently reported HPV 16 and 18 as the predominant subtypes, our findings highlight the presence of other HR-HPV types 58 and 59 among OSCC patients.

## Introduction

Human papillomavirus is an oncogenic virus that is transmitted sexually and is strictly epitheliotropic [1], causing a broad range of diseases such as benign warts or invasive cancers of the mucosal and cutaneous epithelial tissues of the genital area, skin, and throat [2]. HPV infection can be risk factor for development of head and neck squamous cell carcinomas (HNSCC) that includes cancers of the oral cavity, oropharynx, hypopharynx, larynx, and sinonasal tract, including some regions of the upper digestive tract [3, 4]. Globally, HPV is estimated to be the contributing factor for approximately 5% of all cancer cases [5]. HPV has also been reported to be associated with 70% of oropharyngeal squamous cell carcinoma (OPSCC) cases globally [6], but association with oral squamous cell carcinoma (OSCC) remains controversial. Among the 226 known genotypes of HPV, HPV-16 and 18 are considered the major oncogenic types [7, 8]. These high-risk HPV genotypes are associated with the following cancer types: oropharyngeal squamous cell carcinomas and cervical (91%), anal (91%), penile (63%), vulvar (69%), and vaginal (75%) regions [9]. The early HPV genes E6 and E7 encode oncoproteins that contribute to HPV-associated carcinogenesis [10].

OPSCC is the sixth leading cause of cancer worldwide [11]. In recent decades, a surge in oropharyngeal cancer rates has occurred, especially in high-income countries [12]. An estimated 9.0–13.0% of the population worldwide is infected, and 6 million individuals are diagnosed with the disease annually. Approximately 650,555 new cases and 300,000 resulting deaths due to head and neck cancer (HNC) are reported annually [3, 13]. According to US statistics, HPV is among the major etiologies of sexually transmitted infections in the country [14], and recent reports suggest that the percentage of HPV-linked OPSCC cases has increased sharply from less than 20% to above 70% within 20 years in the U.S. and other European countries [15]. However, the prevalence HPV-linked OSCC shows heterogeneity and varies from 0 to 37% worldwide [16]. Studies have found strong evidence connecting oral HPV with various risk factors, such as the alcohol consumption and tobacco [8], changes in sexual behavior (orogenital sexual habits and multiple sexual partners) and a weakened immune system [17].

In the Indian subcontinent, OSCC constitutes a major public health concern, ranking among the top three cancers in the country [18]. As of 2020, 75,290 deaths and 1,35,929 new OSCC cases have been reported [19]. It is the third most common type of cancer among males and females, representing one-third of the global cancer burden [20]. However, there has been inconsistent and sparse information regarding the prevalence and genotype of oral HPV in the general Indian population. *Nandi* et al., through their review article, inferred that the prevalence of HPV-associated HNCs ranges from 0 to 86.6% across India. Majority of the HPV studies from India are on OSCC patients and data on HPV positive OPSCC is limited [21]. The prevalence of HPV associated OSCC in India is estimated to be higher due to additional practice of using smokeless tobacco, betel quid, areca nut, lime paste to name a few. A separate PCR based study on biopsy samples by Bijina et al. [22] and Bhandhary et al. [23] among OSCC patients of Mangalore, Karnataka indicated that HPV prevalence to be 40.4% and 0%, respectively. There exists a discrepancy of results from same geographical location itself. However, the exact prevalence of HPV in HNCs is still unclear; thus, the current study is aimed to detect and identify high-risk HPV genotypes among OSCC and OPSCC patients visiting tertiary care hospitals in Mangalore.

## Materials and methods

### Study population

Patients aged 18 years and older with stage III or IV histologically confirmed HNSCC who underwent elective surgery or provided biopsy sample for diagnostic purpose at the Department of Oral Surgery, A.J. Institute of Dental Sciences, Mangalore, between February and July 2024 and who were willing to consent to their participation in the proposed study were included. A total of 25 patients which included 22 OSCC cases and 3 OPSCC cases were recruited as part of this study.

### Clinical data, sample collection and transportation

Sociodemographic information was gathered from the enrolled patients according to their availability and willingness to respond. All clinical data of the patients were collected from the medical records of the hospital after informing and collecting consent from the patient.

3–5 mm tissue from either surgically removed tumor or from biopsy sample collected for diagnostic purpose was transported in normal saline to the A.J. Research Centre in suitable triple-layer packaging in the cold chain. An aliquot of the sample was transported from the A.J. Research centre to the Manipal Institute of Virology (MIV) in dry ice for genotyping and sequencing. All the samples were aliquoted and stored at -80 °C until further analysis.

### Nucleic acid extraction and polymerase chain reaction (PCR)

HPV DNA was extracted from the processed biopsy samples via a Trueprep^®^ Auto v2 Cartridge-based Universal Sample Preparation Kit (Molbio^®^ Diagnostics Pvt Ltd.). The processing of biopsy sample consisting of pulverising the sample with pestle using lysis buffer provided in the Trueprep^®^ kit. The extracted nucleic acid was tested for high-risk HPV types 16/31 and 18/45 by using a Trunat^®^ HPV-HR kit (Molbio^®^ Diagnostics Pvt Ltd), which has been validated and marketed for testing cervical samples.

Conventional nested multiplex PCR was performed on all samples to amplify the L1 region of HPV via PGMY and MGP primers [24], which are commonly used for genotyping HPV. The first-round assay components used were 25 µL of reaction mix containing 12.5 µL of 2X master mix, 0.25 µL of 10 µM PGMY primer mix, 1 µL of 25X enzyme mix, 6.25 µL of nuclease-free water and 5 µL of template DNA. The cycling conditions consisted of initial denaturation at 95 °C for 10 min, followed by 40 cycles at 95 °C for 1 min, 50 °C for 1 min, and 72 °C for 1 min. An additional extension step was performed at 72 °C for 5 min. The PCR amplicons were subjected to agarose gel electrophoresis and visualised under UV. For the second round of PCR, the reaction mix included a total volume of 25 µL, which was composed of 12.5 µL of 2X master mix, 0.5 µL of 10 µM MGP primer mix, 1 µL of 25X enzyme mix, 10 µL of nuclease-free water, and 1 µL of first-round product. The reaction began with initial denaturation at 95 °C for 10 min, followed by 40 cycles of 95 °C for 1 min, 50 °C for 1 min, and 72 °C for 1 min. A final extension step was carried out at 72 °C for 5 min. The PCR products were visualised under a UV-transilluminator after agarose gel electrophoresis.

### Sanger sequencing

The PCR products (HPV positive only) were purified with a QIAquick gel extraction kit (Qiagen, Germany) since non-specific bands were visualised in gel. The PCR amplicons were then sequenced for the HPV L1 gene via the Big Dye Terminator Cycle sequencing kit (ThermoFisher Scientific, USA) method with MGP primers. The purified sequences thus obtained were then analysed on a 3500XL Genetic Analyser (ThermoFisher Scientific, USA).

### Sequence analysis

Analysis of all the HPV L1 gene sequences obtained from Sanger sequencing was carried out using the Sequencher DNA analysis software (version 5.4.6). The HPV L1 gene sequences obtain were submitted to GenBank with accession numbers: PQ518656, PQ518657, and PQ518658. For phylogenetic analysis, reference L1 gene sequences were downloaded from the NCBI. MEGA (Molecular Evolutionary Genetics Analysis) software version 11 was used to perform phylogenetic analysis via the maximum likelihood method (bootstrap value = 1000).

### Statistical analysis

Statistical analysis of the data was performed by using GraphPad Prism software version 10.4.0 for Windows (GraphPad Software, Boston, Massachusetts, USA). Descriptive data were presented as frequencies and percentages. Paired t-test and Wilcoxon matched-pairs signed rank test were used to evaluate the significant differences and the association between study variables. A p-value of less than 0.005 (< 0.005) was considered statistically significant.

## Results

### Characteristics of the study population

**Fig. 1:**
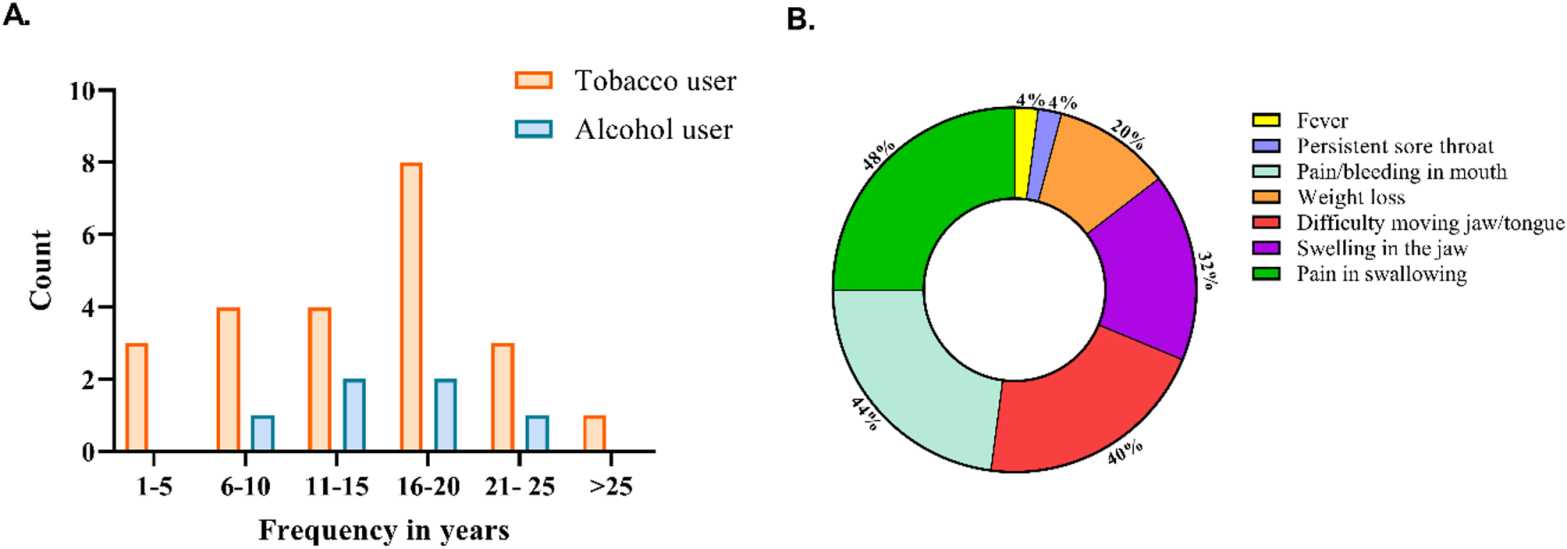
Duration of habit and clinical features of the study population. **A**) Graph representing the duration of habit in years for both alcohol and tobacco. **B**) Pie chart representing the frequency of clinical features of OSCC and OPSCC patients during enrolment in the study

Among the OSCC patients (*n* = 22) and OPSCC patients (*n* = 3), 96% (*n* = 24) were males, and only one patient was female (4%). The patients’ ages ranged from 36 to 80 years (mean = 57.4 years), and 76% (*n* = 19) of the study population had low socioeconomic status. All the patients had a habit history except for one patient with no habit history, and no information was available for one patient (Table 1). The habit of using smokeless tobacco was observed in 32% of the cases, followed by smoking in 28%, and the consumption of both alcohol and tobacco in 28% of the cases. Most of the tobacco users had been using tobacco for 16–20 years (Fig. 1). The alveolus (40%) and tongue (32%) were the most common sites of cancer. Most of the patients presented with complaints of pain while swallowing (48%), pain/bleeding in the mouth (44%), and difficulty moving their jaw/tongue (40%) (Fig. 1).

**Table 1:** Characteristics of the study population

PARAMETERS	CATEGORIES	HPV; *N*= 25					
		Positive (*n* = 3)		Negative (*n* = 22)		Total cases (*n* = 25)	
		Number	%	Number	%	Number	%
**Age**	Mean	51 (45–57)		58.4 (36–80)		57.4 (36–80)	
**Gender**	Males	3	100	21	95.45	24	96
	Females	0	0	1	4.54	1	4
**Socioeconomic status (SES)**	Low SES	2	66.67	17	77.27	19	76
	Middle SES	1	33.33	5	22.73	6	24
**Habit**	Smokeless Tobacco	2	66.67	6	27.27	8	32
	Tobacco smoking (Cigarette and/or Beedi)	0	0.00	6	27.27	6	24
	Smoking and smokeless tobacco	0	0.00	2	9.09	2	8
	Tobacco and alcohol consumption	1	33.33	6	27.27	7	28
	No Habit	0	0.00	1	4.55	1	4
	Information not provided	0	0.00	1	4.55	1	4
**Cancer anatomical sites**	Alveolus	0	0.00	10	45.45	10	40
	Tongue	1	33.33	7	31.82	8	32
	Buccal mucosa	1	33.33	3	13.64	4	16
	Labial mucosa	1	33.33	2	9.09	3	12
	Hard palate	0	0.00	2	9.09	2	8
	Soft palate	0	0.00	2	9.09	2	8

### Detection of high-risk HPV genotypes

**Fig. 2:**
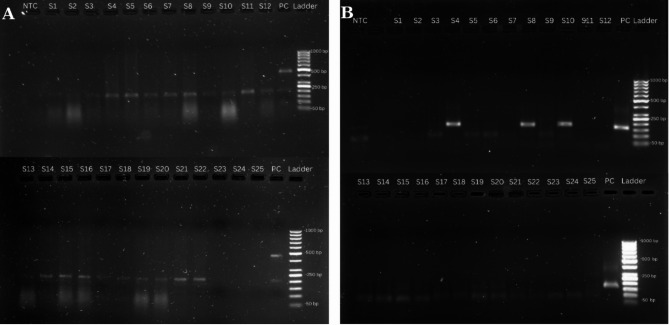
Agarose gel electrophoresis **A**) 1st round of HPV conventional nested PCR using PGMY09/11 primer sets (expected product size~ 450 bp). **B**) 2nd round HPV PCR product using MGP primer sets (expected product size ~ 158–168 bp). Samples 4, 8 and 10 were positive

Among the 25 tumor tissue samples, 0% tested positive for high-risk HPV 16/31 and 18/45 via the Trunat^®^ HPV-HR Kit (Molbio^®^ Diagnostics Pvt Ltd.), whereas 12% (*n*=3) tested positive for other high-risk HPV via nested conventional multiplex PCR (Fig. 2).

**Fig. 3:**
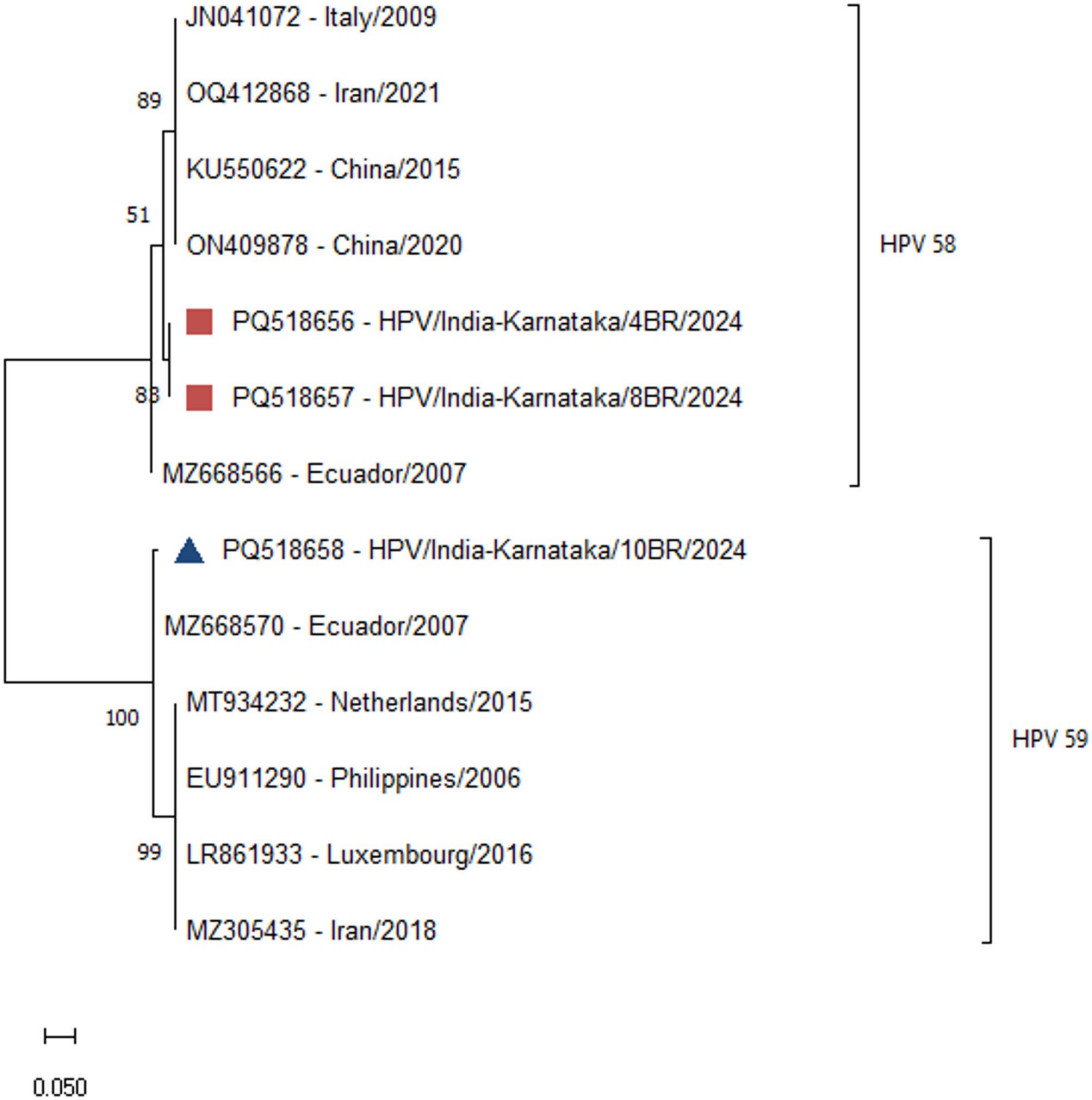
Phylogenetic tree (L1 gene sequence). The sequences from the current study are marked with red boxes (**HPV 58**) and blue triangles (**HPV 59**). The phylogenetic tree was constructed using the maximum likelihood method with a bootstrap value of 1000 via MEGA11

Sequence analysis of the L1 gene revealed that these sequences belonged to the HPV genotypes 58 (*n* = 2) and 59 (*n* = 1). The HPV 58 samples, HPV/India-Karnataka/4BR/2024 (PQ518656) and HPV/India-Karnataka/8BR/2024 (PQ518657) presented the highest similarity (97.09% and 97.14%, respectively) to those from Ecuador. Similarly, the HPV 59 sample, HPV/India-Karnataka/10BR/2024 (PQ518658), presented the highest similarity (99.11%) with the Ecuador strain (Fig. 3).

### Demographic, socioeconomic and behavioural characteristics of HPV-positive and HPV-negative patients

**Fig. 4:**
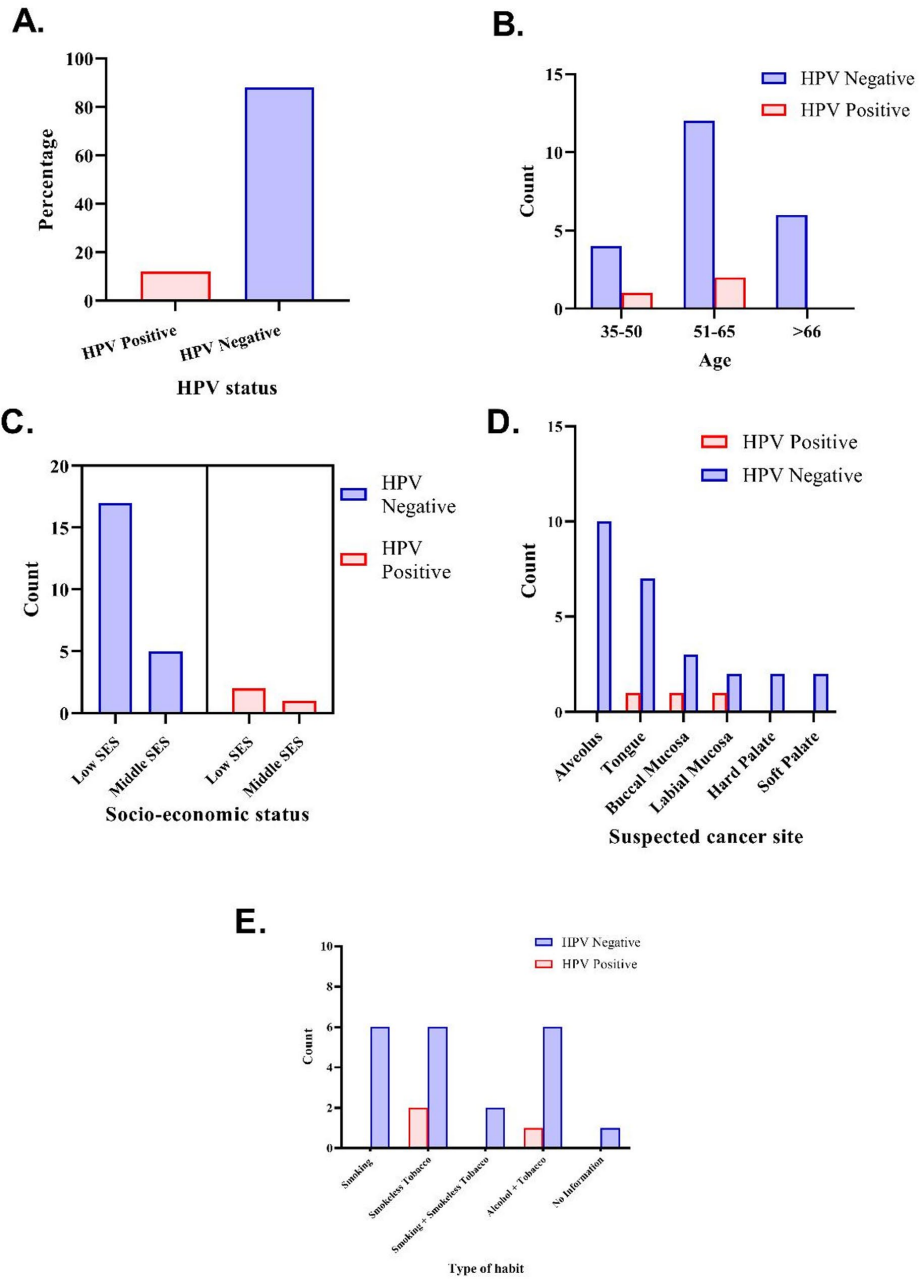
Bar graph showing the associations between HPV-positive and HPV-negative patients. **A**) HPV positivity. **B**) Age distribution. **C**) Socioeconomic status. **D**) Cancer site. **E**) Type of habit. HPV-positive cases are represented by red bars, and negative cases are represented by blue bars

All three HPV-positive patients were OSCC cases who were males, and the mean age of the HPV-positive patients was 50.7 years. However, the mean age of the HPV-negative patients was 58.4 years. Two of the HPV-positive patients had low socioeconomic status, and one had middle socioeconomic status. The majority of the negative cases (77.2%) also had low socioeconomic status. The cancer sites of the HPV-positive patients were oral cavity and included the lateral side of tongue, buccal mucosa and labial mucosa. Furthermore, the cancer site for most of the HPV negative was oral cavity except three cases were cancer was present in the oropharynx and included the soft palate (*n* = 2) and base of tongue (*n* = 1). Smokeless tobacco use was reported in two (66.7%) of the three HPV-positive patients, whereas 27.8% of the HPV-negative patients reported smoking, smokeless tobacco or a combination of alcohol and tobacco (Fig. 4).

## Discussion

The estimated prevalence of HPV-associated HNSCC in India varies between 0% and 87%, depending on the study’s location [21]. In the present study, 12% of cases were positive for oral HPV DNA. However, the sample size included in the current study (*n* = 25) is too small to provide information regarding the actual prevalence rate of HPV associated OSCC among the population. Zhu et al. reported that the detection of HPV DNA in oral specimens may vary broadly according to the technique used, the population under consideration, the type of sample, and the site of the cancer [25]. In the present study, two of the HPV-positive cases were HPV 58, and one was HPV 59. Studies previously conducted in different parts of India have reported HPV 16 or HPV 18 as the common HPV type associated with OSCC [26, 27], and only one study has reported HPV 58 (0.57%) and coinfection with HPV 16, HPV 58, and HPV 82 in HNSCC [28]. HPV 58 infection in oral cavity of patients has been previously reported in other parts of the world [29–31], and the estimated global prevalence of HPV 58 among oropharyngeal cancer patients is 0.29–1.48%, with a prevalence of 2.09% in Asia [32]. Baboci et al. reported the presence of HPV 58 DNA in oropharyngeal tumor tissue, detected HPV 58 RNA encoding oncoproteins, and observed a strong serum antibody response against these oncoproteins [33]. Although HPV 59 infection in oral cancer has not been reported in India, HPV 59-associated cervical cancer has been reported with an incidence rate of 0.28–1% among women from Delhi, India [34]. The global prevalence of HPV 59 infection in oropharyngeal cancer patients is reported to be 0.10–0.88% [32]. In East Asian countries, particularly in China, Hong Kong, Korea and Japan, the prevalence of squamous cell carcinomas associated with high-risk HPV types 58 and 59 is significantly high [35].

As a part of the present study, along with the HPV detection, we also evaluated the sociodemographic, behavioral and clinical parameters of the OSCC and OPSCC patients included in the study. The HPV-positive patients in the current study were in the fourth and fifth decades of life. Similar to our findings, a study conducted by Gholap et al. in Mumbai, India, reported that the rates of HPV positivity were 27.7%, 29.6% and 22.2%, respectively, among individuals aged 40–49, 50–59, and 60–69 years [36]. Numerous studies have shown that the prevalence of HPV-driven OSCC is rapidly increasing and that it occurs predominantly in males rather than females [26]. In accordance with these findings, our study reported that all the HPV-positive patients were males in all the studied age groups. All the HPV-positive OSCC patients in the present study reported a history of either tobacco or alcohol consumption or both. Similarly, previous studies have reported a significant association of HPV-positive HNSCC with high tobacco usage and alcohol consumption [37]. According to previous studies, approximately 60% of Indians use a smokeless form of tobacco, which increases the risk of oral cancer [38]. In our study, 66.6% of the HPV-positive patients used smokeless tobacco. Furthermore, consistent with other studies, in HPV positive cases the tumor was located in the buccal mucosa, labial mucosa and lateral side of the tongue [22]. A study using National Cancer Registry Program (NCRP) data from India reported oropharyngeal cancer as the most common HPV-related cancer in Indian males (63.2%) compared with females (3.6%), highlighting significantly higher infection rates in men compared to women [39]. Thus, the detailed studies are required to gain further insights on the role of HPV in OSCC.

It is important to carry out additional large-scale, in-depth multicentric research on oral HPV, which should include a much larger and more heterogeneous study population in order to identify the actual burden, prevalence and the subtypes of HPV predominantly associated with OSCC in India. These studies need to include methods like the identification of viral RNA and antibodies, and the detailed analysis of differential proteomic expression patterns. Only with such thorough research we can acquire a full and accurate understanding of the role of HPV in oral cancers among various populations, its metastatic potential, activation of associated cellular pathways, and how the virus specific disease interacts with and reacts to multiple treatment modalities. This multidisciplinary strategy will give us essential information about the larger context of oral HPV infection and its implications for public health, vaccination and treatment approaches.

## Conclusion

Our study revealed a notably greater burden of HPV among OSCC patients attending a tertiary care centre in Mangalore. Although PCR-based identification of HPV DNA in tumor tissue is an indication of disease etiology, further studies related to the detection of oncoprotein (E6 and E7), mRNA in tumor specimens or antibodies against oncoproteins are essential to strengthen the current findings. Tobacco and alcohol consumption may be identified as prime risk factors, suggesting a synergistic effect with HPV in OSCC development. Our findings necessitate more prospective studies and further research through larger, multicentric studies to better understand the burden of oral HPV in India and inform effective prevention and control strategies.

## Data Availability

The data that support the findings of this study are available upon request from the corresponding authors.

## Declarations

### Clinical trials registry

Clinical trials registry, India (CTRI Number- CTRI/2024/04/064991) registration was obtained. Study is not a clinical trial however based on IEC recommendations clearance was obtained.
